# The Confluence Point: A New Incision Strategy for Lymphaticovenular Anastomosis in Peripheral Lymphedema

**DOI:** 10.1097/PRS.0000000000012075

**Published:** 2025-03-07

**Authors:** Giuseppe Visconti, Akitatsu Hayashi, Joon Pio Hong

**Affiliations:** Rome, Italy; Kamogawa, Chiba, Japan; and Seoul, Republic of Korea; From 1 UO Chirurgia Plastica, Dipartmento di Scienze per la Salute della Donna, del Bambino e di Sanità Pubblica, Fondazione Policlinico Universitario “A. Gemelli” IRCCS; 2 Breast Cancer Centre, Kameda Medical Center; 3 Asan Medical Center, University of Ulsan.

## Abstract

**Background:**

In the past 5 years, many advances have been made in preoperative planning using new imaging technologies. The high case load of lymphaticovenular anastomosis (LVA) performed using ultra–high-frequency ultrasound led to the discovery of a new incision site—the confluence point—where 2 major functional lymphatic channels merge into one, and then become sclerotic soon after.

**Methods:**

From October of 2021 to May of 2022, 60 consecutive patients with extremity lymphedema who underwent LVA were prospectively assessed. Preoperative planning included indocyanine green lymphography and ultra–high-frequency ultrasound. LVAs at the confluence points were evaluated in terms of operative time and LVA dynamics after the anastomosis, and compared with the incisions without confluence points.

**Results:**

The confluence point was detected preoperatively in 26 cases (43%). The lymphatics proximal to the confluence point showed similar calibers to the distal ones, with no significant size increase, and underwent a lumen obstruction 0.5 to 1 cm after the confluence point in 22 cases (92%). The mean operative time for LVA at the confluence points was 39 ± 8 minutes in upper limb lymphedema and 42 ± 6 minutes in lower limb lymphedema, significantly lower compared with incisions with 2 anastomoses (57 ± 8 minutes for upper limb lymphedema [*P* < 0.0001] and 69 ± 15 minutes for lower limb lymphedema [*P* < 0.0001]).

**Conclusion:**

LVA of confluence points derives from the anatomic findings detectable by ultra–high-frequency ultrasound, and proved to be an effective method to minimize the number of LVAs needed while maintaining the maximal lymph flow and the best dynamics through the anastomosis.

**CLINICAL QUESTION/LEVEL OF EVIDENCE:**

Therapeutic, IV.

Lymphaticovenular anastomosis (LVA) is a minimally invasive and highly effective treatment for lymphedema. Since its introduction by Koshima et al.,^1^ LVA surgery has evolved and diversified rapidly, along with progressive comprehension of the pathophysiology of lymphedema and technologic advances in lymphatic vessels imaging.

The number of open questions that have characterized LVA surgery, including indications for LVA, the role of decongestive therapy, selection of lymphatic vessels, location of incision sites, surgical technique, and configuration of anastomoses, are being progressively solved. The most recent achievements include the preferable choice of overloaded yet high-flow and functional lymphatics,^2,3^ accurate selection of recipient venules,^4,5^ extension of LVA indications to advanced cases,^6–8^ and preference for end-to-end anastomosis^8–10^ with supermicrosurgical technique,^11–13^ in an integrated approach encompassing early surgery and physical therapy, which enhance each other as fundamental components of treatment.^8^ The trend is toward a personalized surgery, focused on each patient’s characteristics, with incision sites unique for each individual and established on the basis of the preoperative selection of propulsive lymphatics and reflux-free venules. Although many questions have been clarified, others are still debated, particularly the site and number of anastomoses needed to achieve a clinical effect.^14^

We introduce a novel method to select the incision sites where lymphatic vessels merge into a single lymphatic channel. This point can be used to perform a single LVA that actually includes multiple major lymphatic channels. This may boost the efficiency of LVA, minimizing the number of LVAs needed while maintaining the maximal lymph flow and the best dynamics through the anastomosis.

## PATIENTS AND METHODS

From October of 2021 to May of 2022, 60 consecutive patients with extremity lymphedema (30 unilateral upper limb lymphedema [ULL] and 30 unilateral lower limb lymphedema [LLL]) who underwent LVA were assessed. Patient demographics are shown in Table 1. The same preoperative planning and surgical technique were used in all cases. All patients underwent lymphoscintigraphy, and the preoperative planning included indocyanine green lymphography (ICG-L) and ultra–high-frequency ultrasound (UHFUS). ICG-L (Fluobean; Fluoptics) was performed with standard technique.^15^ UHFUS (Vevo MD; Fujifilm VisualSonics) was used to locate functional lymphatic channels and favorable recipient venules and plan the skin incisions to perform LVA accordingly.^16–19^ For the purposes of this study, if multiple overloaded lymphatics were located in the selected anastomotic areas, UHFUS was used to visualize their eventual confluence point.

**Table 1.** Demographic Characteristics

Characteristics	Value
No. of patients	60
Mean age (range), yrs	52 ± 14.2 (38–87)
Mean body mass index (range), kg/m^2^	26.3 ± 3.1 (25.2–31.4)
Upper extremity lymphedema, no. (%)	30 (50)
Lower extremity lymphedema, no. (%)	30 (50)
Male sex, no. (%)	6 (10)
Female sex, no. (%)	54 (90)
Preoperative ISL stage, no. (%)	
1	8 (13)
2a	17 (28)
2b	30 (50)
3	5 (9)

ISL, International Society of Lymphology.

### Preoperative LVA Planning

After ICG-L was used to mark the linear, stardust, and diffuse patterns, UHFUS was used to further evaluate the lymphatic channels and recipient venule and, thus, to locate the anastomotic areas.

In case of ICG linear patterns with subsequent lymph extravasation, UHFUS was used to directly visualize the lymphatics and define their characteristics; in case of only stardust or diffuse patterns, UHFUS was used to identify collectors invisible to ICG lymphography. Ultrasound permits identification of functional lymphatics preoperatively, and assessment of their exact position in relationship with the surrounding tissues and structures, as well as their caliber, lumen patency, course, contractility, and degeneration status, in accordance with their histologic features.^3^ Only functional or type I lymphatics according to the UHSUS classification^19^ (normal or ectatic according to the NECST systems,^20^ S0 or S1 according to the lymphosclerosis classification^21^) were considered for anastomosis.

When multiple lymphatic collectors were identified, they were traced by moving the probe upward and downward to trace their course and assess their eventual confluence in a single channel collecting the lymph of all its tributaries. (**See Video [online]**, which demonstrates UHFUS tracing of the confluence point along with the exact point of lymphatic channel degeneration.) We called this point where multiple lymphatic channels converged into a single one the confluence point. At this point, a single LVA can be performed that includes the propulsion of more functional lymphatic channels.

**Video.** This video demonstrates UHFUS tracing of the confluence point along with the exact point of lymphatic channel degeneration.

UHFUS was also used to locate nearby suitable recipient venules, as per our conventional protocol.^5^

The confluence point was never seen with ICG-L because the distance between lymphatic channels is within less than 1 cm, and ICG is not able to provide 2 clear single linear patterns at such close distance (rather, it provides a linear pattern). It is the UHFUS analysis of this linear pattern (when present) that allows understanding of the true lymphatic microanatomy beyond it.

For each patient, the preoperative UHFUS evaluation included the number, location, course, diameter, and degeneration status of lymphatic collectors; the presence of a confluence point; and characteristics of channels afferent to and efferent from the confluence point.

**Fig. 1.**
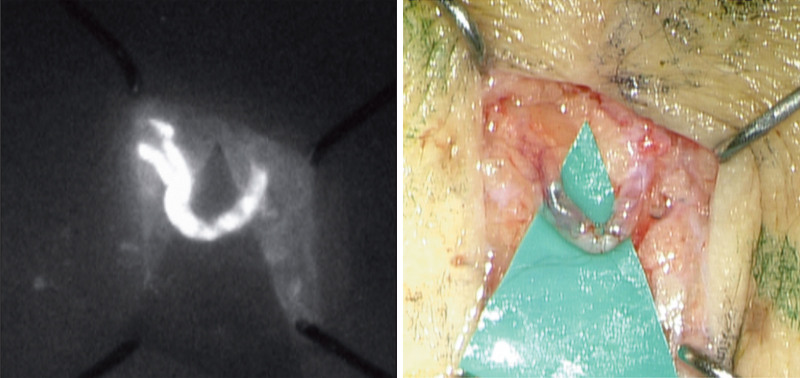
Intraoperative visualization of lymphaticovenular anastomosis at the confluence point under intraoperative microscope–integrated fluorescence mode (*left*) and without fluorescence mode (*right*). Two functional lymphatic channels merge into 1 channel, which is bypassed into a favorable recipient venule.

**Fig. 2.**
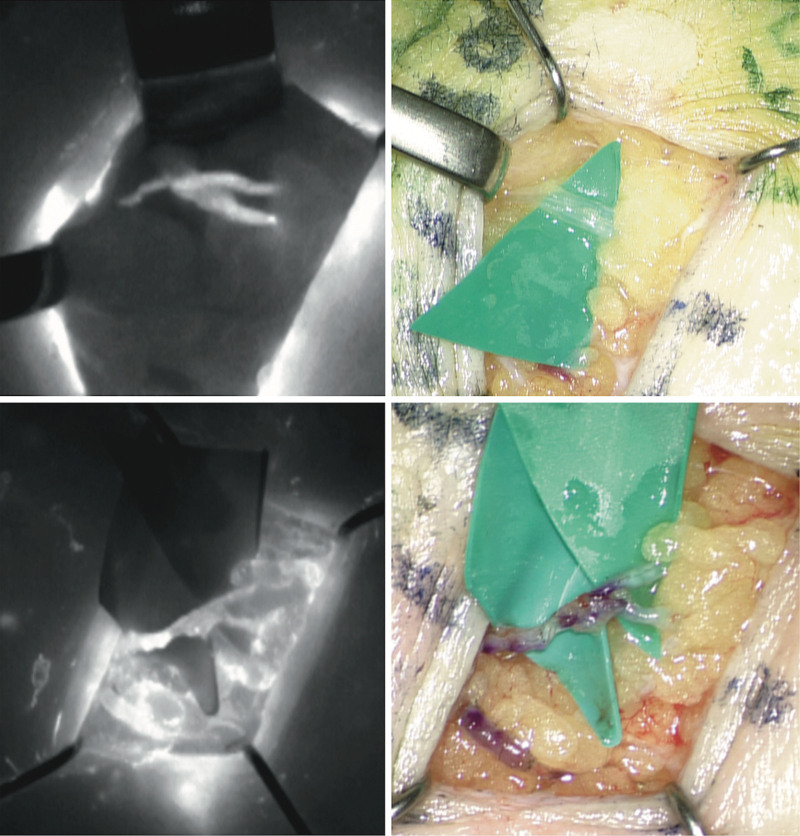
Intraoperative visualization of the confluence point during dissection with intraoperative microscope–integrated fluorescence mode (*above*, *left*) and without fluorescence mode (*above*, *right*). A single lymphaticovenular anastomosis using the single proximal lymphatic channel after the confluence point performed on a favorable recipient venule and shown with intraoperative microscope–integrated fluorescence mode (*below*, *left*) and without fluorescence mode (*below*, *right*).

After skin incision, lymphatic channels were identified under the operating microscope (OPMI Pentero 800; Zeiss) with the ICG fluorescence module and bypassed to the selected venules using 12-0 nylon sutures (S&T; Neuhausen). After the LVA was established, the lymph flow though the LVA was evaluated under the operating microscope both clinically and using incorporated infrared fluorescence (Infrared 800; Zeiss) (Figs. 1 and 2).

Intraoperative findings, including the number and size of the lymphatics, the presence of the confluence point, and the anastomosis outcomes, were recorded.

Categorical variables are presented as numbers and percentages. Continuous variables are presented as means and SD. Quantitative analysis included the detection rate of the confluence point, as well as lymphatics number and caliber before and after their convergence. The chi-square or Fisher exact test was performed for categorical variables, and the independent samples *t* test for continuous variables. The accuracy of UHFUS in detecting the confluence point preoperatively was assessed by using Cohen kappa coefficient to calculate the agreement between the preoperative and intraoperative findings. A study of 128 patients receiving LVA demonstrated that the flow dynamics through the anastomosis has an independent impact on postoperative limb volume reduction (OR 3.32; *P* = 0.02), need for a compression garment (OR 2.54; *P* = 0.02), reduction of compression class (OR 2.82; *P* = 0.01), and the composite outcome (OR 3.32; *P* < 0.01).^4^ Therefore, for the purposes of this study, every LVA was classified according to the flow dynamics as backflow, slack, or outlet type, each one corresponding to a poor, mediocre, or good outcome, respectively, of the procedure.^9^ All the LVAs were performed in an end-to-end fashion.

LVAs at the confluence points were evaluated in terms of operative time and association with good (outlet), mediocre (slack), or poor (backflow) LVA dynamics after the anastomosis, and compared with the incisions without confluence points.

## RESULTS

The 60 patients included 30 cases of ULL (all secondary to breast cancer treatment) and 30 cases of LLL (all secondary to pelvic cancer or melanoma treatment). Linear patterns on ICG-L were found in 41 patients (68%), including all the patients with International Society of Lymphology (ISL) stage 1 or 2a and 16 with ISL 2b. ICG-L was not able to show the confluence point. Lymphatic collectors suitable for anastomosis were detected preoperatively in all cases, and in 12 patients (29%), more than 1 lymphatics were identified below a single linear pattern.

**Fig. 3.**
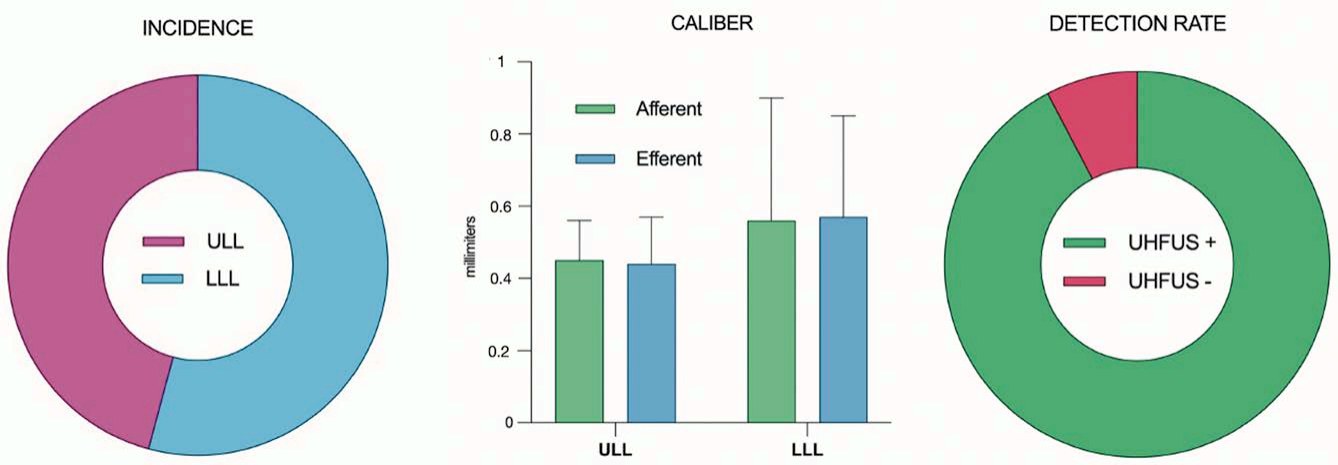
(*Left*) Incidence: the confluence point was found, with similar rates, in 11 ULL cases (17%) and 13 LLL cases (15%). (*Center*) Caliber: the afferent and efferent collectors of the confluence point showed similar calibers, with no size increase after the convergence, in both ULL and LLL. (*Right*) Detection rate: the preoperative detection of the confluence points by UHFUS was confirmed intraoperatively in 24 of 26 cases, with substantial agreement (k = 0.93 [95% CI, 0.838 to 1.000]) between the preoperative and intraoperative findings.

The confluence point was detected preoperatively in 26 cases (43%), and a single confluence point was identified per patient, always located distal to the elbow for ULL and distal to the knee for LLL. In 9 patients (15%), 2 nearby and never-merging lymphatics were identified, and these were scheduled for 2 separate LVAs at the same incision site. No statistical difference was found for the detection rate of confluence points at different ISL stages of disease. Intraoperative exploration confirmed the presence of the confluence point in all except 2 cases, in which the collectors attached one to the other, but they never merged within the incision site, showing substantial agreement between the preoperative and intraoperative findings (k = 0.93 [95% CI, 0.838 to 1.000]) (Fig. 3).

The average size of collectors afferent to the confluence point was 0.45 ± 0.5 mm in ULL and 0.56 ± 0.34 mm in LLL, similar to the parallel nonmerging lymphatics, which measured 0.46 ± 0.13 in ULL and 0.54 ± 0.22 in LLL. The lymphatic proximal to the confluence point showed similar calibers to the distal ones, with no significant size increase, and underwent a lumen obstruction after 0.5 to 1 cm after the confluence point in 22 cases (92%).

On intraoperative fluorescence, the lymphatics proximal to the confluence point were more fluorescent than the distal ones in all cases, and by alternatively clamping the distant vessels it was possible to confirm the flow to be the resultant sum of the afferent ones.

The total number of incision sites was 66 for ULL and 86 for LLL. The total number of LVAs was 77 for ULL and 104 for LLL, with an average number of 2.48 LVAs for each case of ULL (range, 1–5) and 3.35 LVAs for each case of LLL (range, 1–7). There were 24 confluence LVAs: 13 for ULL (14%) and 19 for LLL (15%).

All 24 confluence point LVAs, considered separately, were associated with a good (outlet) outcome; conventional LVA (*n* = 157) showed a good (outlet) outcome in 148 cases (94%); and a mediocre (slack) or poor (backflow) outcome occurred in 9 cases (6%), with no significant difference (*P* = 0.6).

In ULL, there was, in the same incision, 1 LVA in 45 sites (68%), 1 confluence LVA in 11 sites (17%), and 2 LVAs of 2 separate lymphatics in 10 cases (15%). In LLL, there was 1 LVA in 1 incision in 56 sites (65%), 1 confluence LVA in 13 sites (15%), 2 LVAs of 2 separate lymphatics in 15 cases (17%), and 3 LVAs of 3 separate lymphatics in 2 sites (3%).

**Fig. 4.**
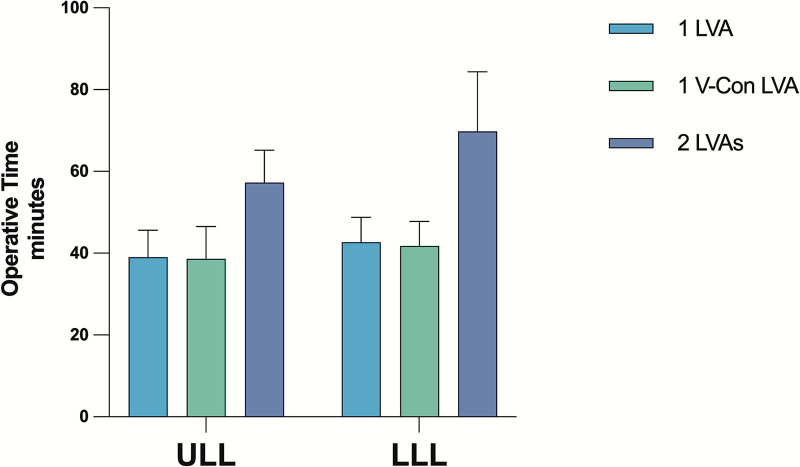
Comparison of operative time for single-incision procedures according to anastomosis type and number. The operative time of LVA of merging lymphatics at the confluence (*V-Con*) point is similar to single LVA for both ULL (40 ± 6 versus 39 ± 8 minutes) and LLL (43 ± 7 versus 42 ± 6 minutes), but is significantly shorter compared with incisions with 2 anastomoses (57 ± 8 minutes for ULL [*P* < 0.0001] and 69 ± 15 minutes for LLL [*P* < 0.0001]).

The operative time for single-incision procedures was on average 42 ± 10 minutes in ULL and 49 ± 14 minutes in LLL. Separately considering the confluence LVA, the mean operative time was 39 ± 8 minutes in ULL and 42 ± 6 minutes in LL, significantly lower compared with the incisions with 2 anastomoses (57 ± 8 minutes for ULL [*P* < 0.0001] and 69 ± 15 minutes for LLL [*P* < 0.0001]) (Fig. 4).

## DISCUSSION

The efficacy of LVA strictly relies on the selection of high-flow functional lymphatics and suitable reflux-free recipient venules. Preoperative lymphatic selection for LVA has been a focus of interest, and was the driving force for advances in understanding of lymphedema anatomy and pathophysiology.

ICG-L has traditionally been the most frequently used tool for preoperative selection of lymphatic vessels, but is now recognized as unable to stand alone in preoperative LVA planning; therefore, other techniques have been introduced progressively.^9^ ICG-L cannot detect the lymphatics in deep layers or in areas masked beneath the dermal backflow (in most moderate to advanced cases), and is contraindicated in patients with iodine allergy.^3^ Our experience with UHFUS allowed us to overcome the limitations of ICG-L and to incorporate the anatomic acquisitions in LVA planning.

It is a common mistake to compare the lymphatic and the venous system.^22^ The lymphatic vessels are more independent than veins, with fewer interconnections and branching at narrow angles, and there is no bridge between the superficial and the deep lymph vessels. Moreover, veins increase in diameter in the proximal regions, whereas lymphatic vessels keep uniform in size between the distal and the proximal regions close to the lymph nodes. Compared with the arterial and venous system, the superficial lymphatics consist of a only horizontal wavy network, as they do not overlap, but are arranged in a plane.^23,24^ Studies on both the upper and lower extremities revealed that each superficial lymphatic collector runs in a straight path toward its corresponding lymph node. These vessels, during their course, converge and diverge, demonstrating their interconnections, which are fewer than those found in blood vessels.^22–26^

In this article, we show the accuracy of UHFUS in detecting these convergence points of lymphatic vessels, and how this can be applied to the selection of anastomosis site and configuration. We found confluence points in 43% of cases; the reasons behind this may be multifactorial, related to lymphatic degeneration, microanatomic variation, and the inability of UHFUS to locate tiny confluence points.

Lymphatic microsurgeons face the situation of multiple lymphatics to be deviated in the same site, and the possibility of not having the necessary recipient venules. This can also happen unexpectedly, because more than 1 lymphatic vessel can correspond to a single linear pattern on ICG-L. Identification of the confluence points allows bypass of all the lymphatics that need to be anastomosed, minimizing the number of LVAs and, consequently, of adequate recipient venules. In case of multiple lymphatic channels to be bypassed in the same incision site, the surgeon must find the same number of adequate recipient venules, or perform complex anastomosis configurations that may not work adequately, jeopardizing the efficacy of the bypass.

This method is particularly useful in areas typically devoid of veins, such as the posterolateral forearm, the anterolateral upper half of the leg, and the lateral thigh.

The confluence point method represents a solution to avoid anastomoses of multiple lymphatic vessels to the same recipient venule, and to avoid the need for complex LVA configurations while maintaining maximal flow through the LVA.

End-to-end anastomosis of one lymphatic channel to one venule has been reported to have the highest long-term patency rate^9^ and the best dynamics^5^ compared with other configurations, as well as compared with intussusception^25^ of multiple lymphatics in a single vein.^9^ By using the confluence point method, it is possible to establish a single end-to-end anastomosis that collects the lymph of many afferent collectors, which finally converge into the confluence, thus allowing bypass of multiple lymph flows into the same recipient venule to maximize lymphatic pressure through the LVA.

Our results showed that the operative time of the confluence point incisions was significantly shorter than for incisions with a similar number of bypassed lymphatics because of the decreased number of anastomoses needed to obtain the same result in terms of anastomosis.

Although this incision strategy enhances surgical efficiency, it cannot be speculated that surgical outcome of performing anastomosis of the confluence point is superior in terms of efficacy compared to patients in whom no confluence point anastomosis has been performed. In all cases, we anastomose the maximum amount of functional lymphatic found at preoperative planning. If a confluence point can be found, a single anastomosis can be performed rather than multiple anastomoses if the incision is not precisely planned on the confluence point (ie, a few centimeters below). In this perspective, we can optimize anastomosis configuration and insetting as well as venule choice, rather than needing to perform acrobatic anastomoses that may increase the rate of anastomosis failure.

The findings of this report are in accordance with recent anatomic studies showing both the convergence of lymphatic collectors and the maintenance of the same caliber of the afferent and efferent lymphatics before and after their confluence.^22–24^

In most cases, the lymphatic collectors underwent obstruction right after the confluence point. It can be speculated that the intraluminal pressure at the confluence point is higher, especially in cases of overloaded lymphatic channels, as in patients with lymphedema. This increased intraluminal pressure may accelerate the degenerative cascade of lymphatic vessels, increasing the likelihood of sclerotic degeneration in the higher pressure points, such as the confluence point. Bypassing the lymphatic channels after they merge in a single lymphatic channel after the confluence point and before it becomes sclerotic allows potential salvage of the entire lymphatic channels tree merging into the confluence point from further progressive degeneration. The LVA is thus performed at the last point before degeneration of the channel, making the confluence point potentially the ideal incision site because the entire functional afferent lymphatics tree is left untouched. This precise point requires careful planning of the recipient venule and surgical strategies as described elsewhere^4,5,26^ to have an optimal recipient venule near the confluence point.

## CONCLUSIONS

Lymphatic confluence points derived from the anatomic findings detectable by UHFUS, and they represent functional points as affected by lymphatic degeneration, likely related to increased intraluminal pressure. Using the confluence point was an efficient method that permitted LVA to be performed at the last point before degeneration, leading to salvage of the entire lymphatic tree beyond and minimizing the number of LVAs while maintaining maximal lymph flow and the best dynamics through the anastomosis. Preoperative detection of the confluence point using UHFUS may enhance the efficiency of LVA surgery.

## DISCLOSURE

The authors have no conflicts of interest to disclose.
